# Machine learning in a time of COVID-19 - Can machine learning support Community Health Workers (CHWs) in low and middle income countries (LMICs) in the new normal?

**DOI:** 10.7189/jogh.11.03017

**Published:** 2021-01-16

**Authors:** Mats Stage Baxter, Alan White, Mari Lahti, Tiina Murto, Jay Evans

**Affiliations:** 1 Centre for Medical Informatics, Usher institute, The University of Edinburgh, Edinburgh, UK; 2 Institute of Applied Health Science, University of Aberdeen, Aberdeen, UK; 3 Health and Well-being, Turku University of Applied Sciences, Turku, Finland; 4 Global Health Academy, Usher Institute, The University of Edinburgh, Edinburgh, UK; 5 Interactive Health Ltd, Inverness, UK

Writing in the Lancet, Schwalbe and Wahl put forward this view:

“Concurrent advances in information technology infrastructure and mobile computing power in many low and middle-income countries (LMICs) have raised hopes that artificial intelligence (AI) might help to address challenges unique to the field of global health and accelerate achievement of the health-related sustainable development goals (SDGs)” *([*1*], p. 1579).*

The application of artificial intelligence (AI) and specifically machine learning (ML) tools seemingly are set to transform global health care in a myriad of ways. They have the potential not only to optimise existing resources, but also to improve health care delivery and outcomes in LMICs. Indeed in 2019, the United Nations Secretary General's High-Level Panel on Digital Cooperation recommended that:

“…by 2030, every adult should have affordable access to digital networks, as well as digitally-enabled financial and health services, as a means to make a substantial contribution to achieving the SDGs” *([*2*], p. 7).*

Vuong et al. [3] cite this same high-level panel as having the view that ML can play an integral part in improving primary care, service delivery, integration and analysis of medical data, as well as responses to epidemics and other medical emergencies. In the context of most LMICs, the burden of implementing front line primary care and service delivery falls squarely on the shoulders of underqualified and underpaid Community Health Workers (CHWs).

Now in a time of COVID-19, the provision of primary care and service delivery in most LMICs has surpassed crisis point. It is likely that this will be further exacerbated as the severe economic downturns in developed countries result in a reduction of state aid to LMICs. Perhaps China, with its expansionist economic interests in Sub Saharan Africa (SSA) and now in the post pandemic era, Latin America, will step into the breech. Whatever the macro geopolitical and economic future holds, it is certain that it is these overburdened CHWs who will be the foot soldiers in the grassroots struggle to prevent a regression in the provision of primary health care, quite apart from aspirations to meet the SDGs.

**Figure 1:**
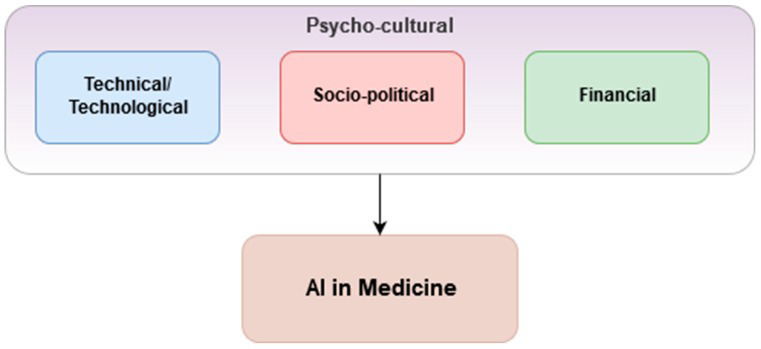
Considerations for successful applications of AI &ML [3].

Prior to the onslaught of COVID-19, Vuong et al. [3] proposed a framework around three considerations within local psycho-cultural contexts which should be in place for the successful application of AI and ML in health care (**Figure 1**). They describe the psycho-cultural context as a phenomenon in which public trust is essential. This, though, is often lacking due to mistrust of the health data safekeeping capabilities and capacities of responsible organisations and governments. If public distrust and political aftershock occur, its re-establishment can be very difficult and costly [4].

Similarly, a low receptiveness among Health Care Workers (HCWs) toward ML tools due to fear of redundancy, disagreement with ML outcomes, lack of technical insight or disruption of workflows, can pose a great challenge to the uptake of ML tools [5].

These “three considerations” are still valid but in the context of a grassroots struggle taking place in many very different local psycho-cultural contexts, they are at a remove from the high level outcomes that Vuong et al. [3] envisaged. Consider for example a female CHW in an informal settlement in Nairobi. Anecdotally, she may already have had to resort to prostitution to purchase medicines for her patients. An ML enabled smartphone would almost certainly help her to make informed clinical decisions but first she would need to be able to afford to pay for the cost of receiving that data. As LMIC economies implode in the wake of the pandemic, the cost of mobile phone data may become the major barrier to implementing what otherwise would be cost-effective and highly beneficial ML solutions. As such, there is already a grassroots financial barrier.

Additionally, while entry costs are comparatively low for implementing simpler ML tools that address repetitive, time-consuming, administrative processes, the stakes and costs increase dramatically when developing ML tools that support clinical care or population health processes. Such costs could negatively affect health care outcomes and patient safety. Technical complexity, computational infrastructure (not least that the AI that underpins ML requires extensive “training”), staff talent required and the organisational capacity and tolerance for risk, all affect the financial cost of implementing and scaling such solutions especially in LMICs. In addition, there is currently a paucity of evaluations of the cost-effectiveness and return on investment of ML tools. In turn, this creates uncertainty among governments looking for improved health care performances and efficiency in return for their investment [6].

Vuong et al. [3] cite another of their considerations as being socio-political. As ubiquitous data collection becomes the norm, so do security, privacy and ethical issues related to storing and sharing data for ML purposes. For instance, not having a clear global consensus regarding a consent framework (eg, GDPR) to regulate the sharing of sensitive data can be a major obstacle [4]. Immediately, trust becomes an issue and another barrier to be overcome by CHWs if they are to win the trust of the communities in which they both live and serve.

Moreover, in many LMICs, members of a small political and economic elite often own the national mobile communication and data and handling companies. Corrupt business practices are often the norm. As such, key challenges revolve around inhibiting the monetisation of sensitive health data preventing it from being purchased by third parties or leveraged to further companies’ own strategic gains. This is an oft-misused business model to overcome initial financial burdens associated with implementing ML tools. It is in clear breach of most national data protection laws [4].

**Figure:**
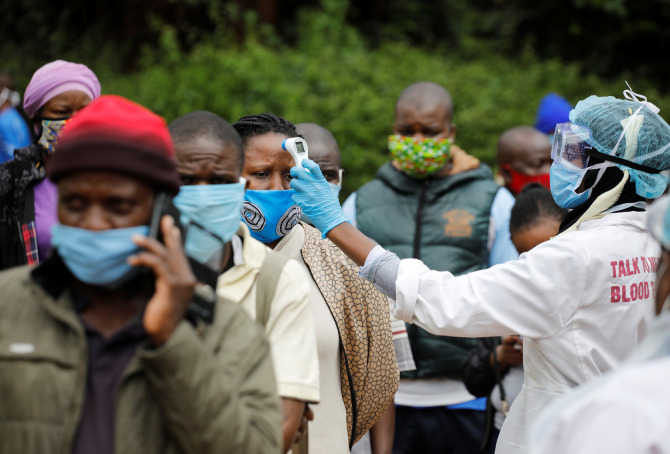
Photo: Source: Adobe Stock Library (Reference: Copyright Reuters, Baz Ratner).

From a regulatory perspective, ML tools should first and foremost be certified in advance of a large-scale implementation to ensure that they live up to a certain degree of safety and effectiveness. However, a reoccurring issue remains in determining such criteria from a regulatory perspective being as ML tools are evolving and improving at a staggering pace [7]. As ML tools continue to evolve so too do regulations. This creates fluid regulatory ecosystems that impede the scale-up of ML tools as regulations might differ across geographical areas and jurisdictions [7].

Lastly, clear legal guidance and protocol on which entity holds liability when malpractice cases arise is currently lacking, which can lead to severe legal and financial repercussions [7].

Furthermore, integrating data aggregation from disparate sources, and standardising it for ML use remains a substantial challenge even in high-income countries. Storing such large volumes of aggregated health data also requires considerable computational infrastructure to facilitate rapid data retrieval based on ML demands. This remains a considerable issue, as storage and retrieval can be computationally and financially costly and time-consuming [8].

While much emphasis has been put on the potential benefits of ML tools in global health, in-depth analysis and evaluations regarding how to best implement and scale ML within existing health care systems, especially within LMICs, are severely lacking. This leaves governments without guidance.

Increased collaboration and coordination between government entities, private sector organisations, civil society and academic communities will be paramount in providing the evidence and guidance needed to properly deploy and scale ML tools within national health systems. In such environments, flexible and scalable open-source software platforms can be utilised to reduce development costs. Similarly, collective focus should be placed on platforms that standardise data harmonisation. Using such platforms could further enhance the interoperability and scale of ML, while also reducing financial entry-level costs [8].

Likewise, international and national law reforms need to be agreed upon regarding data privacy and codes of conduct, possibly adding complementary amendments to already existing protection of health information legislation, while third party usage of health data should be audited and assigned to appropriate authorities [9]. Moreover, multi-disciplinary clinical, operational and legal committees could be formed to oversee large-scale ML implementations to mitigate legal barriers, disruptions of HCW workflows and potentially harmful results of ML outputs. These should be accompanied by an increased focus on building the capacity of ministries of health and government to properly design, deploy, regulate and scale ML tools within their respective heath systems.

Now, in the time of COVID-19, such measures are even more important. If the promises held out for the universal acceptability and efficacy of ML and person-centric health care are to be realised, ML must be affordable at a grassroots level in LMICs. More collaboration and co-design involving mobile health developers, mobile telecoms companies, governments, trans-national organisations and non-governmental organisations (NGOs) could result in affordable generic open-source templates. Most importantly, such a collaboration needs to be done in parallel with paying CHWs a living wage. Achieving such a goal would remove the need for any CHW ever having to resort to prostitution to purchase medicines in order to treat their patients.
